# Explainable Patient-Level Cognitive Impairment Screening via Temporal, Semantic, and Psycholinguistic Multimodal AI

**DOI:** 10.3390/jintelligence14040066

**Published:** 2026-04-15

**Authors:** Abdullah, Zulaikha Fatima, Miguel Jesús Torres Ruiz, Osvaldo Espinosa-Sosa, Carlos Guzmán Sánchez-Mejorada, Rolando Quintero Téllez, José Luis Oropeza Rodríguez, Grigori Sidorov

**Affiliations:** 1 Center for Computing Research, Instituto Politécnico Nacional, Mexico City 07738, Mexico; abdullah2025@cic.ipn.mx (A.); espinosa@cic.ipn.mx (O.E.-S.); cmejorada@cic.ipn.mx (C.G.S.-M.); quintero@cic.ipn.mx (R.Q.T.); joropeza@cic.ipn.mx (J.L.O.R.); sidorov@cic.ipn.mx (G.S.); 2 Department of Computer Sciences, Bahria University, Lahore 54600, Pakistan; 3 Faculty of Allied Health Sciences, Superior University, Lahore 54000, Pakistan; su91-bmitm-f23-248@superior.edu.pk

**Keywords:** cognitive impairment, Alzheimer’s disease, mild cognitive impairment, longitudinal clinical notes, psycholinguistic biomarkers, temporal progression, semantic graph reasoning, explainable AI

## Abstract

Early diagnosis of cognitive decline is vital for timely treatment of mild cognitive impairment (MCI) and Alzheimer’s disease (AD), yet standard clinical assessments often miss subtle longitudinal language changes. We propose a hierarchical hybrid intelligence framework integrating long-context language modeling, temporal progression, semantic graph reasoning, psycholinguistic biomarkers, and contrastive progression learning to classify patient states (Normal, MCI, AD) from longitudinal electronic health record (EHR) notes. The model was trained on 4500 patients and 68,000 clinical notes from Medical Information Mart for Intensive Care III (MIMIC-III) and externally validated on the Medical Information Mart for Intensive Care IV (MIMIC-IV) clinical notes dataset (5200 patients, 72,000 notes). Inputs combined Biomedical and Clinical Bidirectional Encoder Representations from Transformers (BioClinicalBERT) embeddings, Bidirectional Long Short-Term Memory (Bi-LSTM) temporal encodings, Graph Sample and Aggregate (GraphSAGE)-based Unified Medical Language System (UMLS) concept graphs, and psycholinguistic vectors (lexical diversity, grammatical complexity, discourse coherence). On the MIMIC-III hold-out set, the model achieved 99.999% accuracy, a macro F1-score of 0.999, a Receiver Operating Characteristic Area Under the Curve (ROC AUC) of 0.999, and a temporal stability variance of 0.0008. Monte Carlo cross-validation (10,000 folds) yielded 99.997±0.003% accuracy and 0.999±0.001 macro F1. Feature ablation confirmed distinct gains from temporal, semantic, and psycholinguistic modules, improving performance by 1.1% over text-only baselines. Cross-cohort zero-shot testing on MIMIC-IV showed strong generalization with minimal decline in macro F1 and balanced accuracy. Explainability analyses, such as SHapley Additive exPlanations (SHAP) token/concept attribution, attention maps, counterfactual perturbations, and psycholinguistic importance, revealed clinically interpretable markers, such as pronoun overuse, reduced lexical diversity, and syntactic simplification, as predictors of decline. Our framework supports scalable, non-invasive early screening in a variety of healthcare settings by providing longitudinally stable predictions.

## 1. Introduction

A serious public health concern is the rapidly rising prevalence of Alzheimer’s disease (AD) and related cognitive disorders, such as mild cognitive impairment (MCI) (Lee, 2023; Tahami Monfared et al., 2022). An estimated 57 million people worldwide suffered from dementia in 2021, with more than 60% of those affected living in low- and middle-income nations (Antwi, 2023; Livingston et al., 2024; World Health Organization, 2021). By 2050, this number is expected to have nearly doubled every 20 years, to reach 139 million. About 7.2 million Americans aged 65 and older suffered from Alzheimer’s dementia in 2025, and by 2060, that number is expected to rise to 13.8 million. Despite its prevalence, MCI is significantly underdiagnosed (up to 92% of cases go undiagnosed), which restricts the chances for early intervention (Antwi, 2023; Livingston et al., 2024; World Health Organization, 2021).

The significant medical and socioeconomic effects of cognitive impairment are highlighted by epidemiological studies (Wang et al., 2023). Because of its increasing prevalence and the need for long-term care, the global costs for dementia alone is predicted to double by 2030. Cognitive decline affects everyday living, raises caregiver stress, and lowers quality of life, in addition to memory and executive dysfunction (Bruijs, 2025). Because MCI progresses differently and 10–15% of cases turn into dementia each year, longitudinal monitoring is essential (Rajagopal et al., 2024). Healthcare disparities make these issues worse, especially in areas with few diagnostic resources (Rad, 2025). All of these elements work together to highlight the need for non-invasive, scalable, and affordable methods of early detection and ongoing monitoring.

Rather than providing a comprehensive epidemiological or molecular review of Alzheimer’s disease, this study focuses on language as a scalable, non-invasive proxy for longitudinal cognitive decline. Subtle changes in lexical diversity, syntactic complexity, and discourse coherence emerge early in neurodegeneration and can be captured continuously through routinely collected clinical narratives, offering a complementary signal to traditional biomarkers.

Alzheimer’s disease and related disorders arise from progressive neurodegenerative processes that begin years before overt symptoms become clinically apparent, including synaptic dysfunction, network disruption, and established molecular risk factors (J. Li et al., 2025; A. Rahman et al., 2025; Roychowdhury et al., 2025). These long preclinical phases complicate timely identification using conventional biomarkers alone and motivate the exploration of complementary, non-invasive behavioral signals (Gaubert, 2025; Zou et al., 2025).

Although earlier recognition of decline may support clinical planning, standard assessments such as neuropsychological testing, neuroimaging, and cerebrospinal fluid analysis remain costly, episodic, and difficult to scale (Alotaibi & Alharbi, 2025; Taiyeb Khosroshahi et al., 2025; Vinukonda & Jagadesh, 2025). Language captured in routine clinical documentation offers an alternative observational source that may contain indirect markers of cognitive change, including reduced lexical diversity, syntactic simplification, and impaired discourse coherence (Abdullah et al., 2025c, 2024). The increasing availability of electronic health record (EHR) narratives therefore provides an opportunity to study these signals longitudinally as research proxies for cognitive trajectories rather than as standalone diagnostic or screening tools (Abdullah et al., 2025b).

Clinicians routinely document orientation, memory, attention, and functional capacity using descriptive narrative language; consequently, documentation implicitly encodes cognitive status. We therefore treat clinical language as an indirect observational proxy of cognition rather than a direct neuropsychological measurement.

Language disturbances are commonly observed in Alzheimer’s disease and mild cognitive impairment and have been associated with distributed changes in memory, executive function, and attentional networks. Prior work reports reduced lexical diversity, word-finding difficulty, syntactic simplification, and impaired discourse coherence in affected individuals, suggesting that longitudinal language patterns may reflect aspects of cognitive status. Accordingly, language can be viewed as a non-invasive and indirect behavioral proxy of cognitive function rather than a direct measure of neurodegeneration, motivating the study of longitudinal linguistic biomarkers.

Despite advances in clinical natural language processing (NLP), including Transformer-based architectures, most existing approaches analyze notes cross-sectionally or in isolation, limiting their ability to capture within-patient temporal progression. Additional challenges include semantic drift in medical terminology, limited learned representation interpretability, and reduced generalization across documentation styles and institutions.

The clinical gap addressed in this work is therefore methodological: the absence of scalable approaches to separate potential longitudinal cognitive signals from broader documentation variability in complex care environments. Because ICU notes are authored by clinicians and may reflect clinician perception, illness severity, or care intensity in addition to cognition, we model language trajectories as documentation-based proxies rather than definitive indicators of impairment. Our framework focuses on characterizing within-patient temporal patterns to study whether progressive changes can be distinguished from transient fluctuations related to staffing, acuity, or note style. To address these gaps, we present a hierarchical, multimodal hybrid architecture for patient-level cognitive state prediction that incorporates:

(a) Long-context language modeling using Longformer to capture distributed linguistic cues within lengthy clinical notes.
(b) Temporal progression modeling using Bidirectional Long Short-Term Memory (Bi-LSTM) with attention to track cognitive deterioration in sequential patient encounters.
(c) Clinical semantic graph reasoning using Graph Neural Networks on UMLS concepts to model shifts in the co-occurrence of medical concepts over time.
(d) Interpretation of psycholinguistic biomarkers, including lexical diversity, syntactic complexity, and discourse coherence, to provide clinically actionable insights.
(e) Contrastive learning to enhance sensitivity to subtle progression within the patient.

Trained on MIMIC-III and externally validated in MIMIC-IV, the hybrid model, integrating embeddings as well as temporal, semantic, and psycholinguistic characteristics, outperformed baseline approaches. SHAP, attention, and linguistic analyses provide non-invasive interpretable digital biomarkers for scalable cognitive monitoring. The remainder of the paper is structured as follows: Section 1 describes Related work and introduction, Section 2 details the methodology, Section 3 presents results, Section 4 discusses findings, and Section 5 concludes.

For related studies and background knowledge, we are exploring existing methods such as the following: Early diagnosis of AD is challenging due to progressive neurodegeneration and subtle biomarkers in the early stages. Mohammed et al. combined GBM and DNN on 35 demographic, behavioral, and clinical features, achieving 92.6% accuracy and an AUC of 0.94 (Mohammed et al., 2025). Paduvilan et al. used an attention-driven SVM-DNN ensemble for neuroimaging-based classification, improving small-sample sensitivity to 98.5% accuracy (Paduvilan et al., 2025). Zhang et al. proposed OpDoctornet with MAMBA-based feature extraction for attention-driven neurodegenerative screening (Zhang et al., 2025), while Alayba et al. fused multi-CNNs with handcrafted MRI descriptors, reaching 98.8% accuracy (Alayba et al., 2024). Slimi et al. showed CNN-SNN hybrids maintain temporal modeling, dropping accuracy from 99.58% to 75.67% without SNN (Slimi et al., 2025). Zolfaghari et al. combined dual CNN ensembles with SVM, RF, and KNN, achieving 99.06% accuracy (Zolfaghari et al., 2025), and Akindele et al. integrated ResNet-50, CBAM, and multi-head attention to reach 99.17% accuracy (Akindele et al., 2025). Vinukonda and Jagadesh combined DeepLabv3+ segmentation, LENET-5 features, and ERESNEXT classifiers, attaining 98.12% accuracy (Vinukonda & Jagadesh, 2025).

Nour et al. applied deep ensemble learning with five 2D-CNNs for EEG-based classification, achieving 97.9% accuracy (Nour et al., 2024). Alorf proposed a transformer-CNN hybrid for six-stage AD classification on rs-fMRI, achieving 96% multiclass and 98.36–99.65% binary accuracies (Alorf, 2025). Aghdam et al. highlighted low repeatability in neuroimaging-based ML models, stressing robust preprocessing and cross-cohort validation (Aghdam et al., 2025). Ahamed et al. combined hybrid filtering with EfficientNetV2B3 and Grad-CAM++ for interpretable early detection at 99.45% accuracy (Ahamed et al., 2025).

In terms of privacy-preserving EEG-based hybrid fusion models, Umair et al. achieved 97.1% accuracy with <1 MB memory across 88 participants (Umair et al., 2025). Using stacking ensembles (Javeed et al.) and DL models with SMOTE, Rahman et al. showed the importance of architecture selection and data balancing, with InceptionV3 achieving 98% accuracy (Javeed et al., 2024; M. S. Rahman et al., 2025). Alotaibi and Alharbi combined MRI preprocessing, feature selection, and wavelet neural networks with ISSA optimization to reach 95% accuracy (Alotaibi & Alharbi, 2025). Zanola et al. proposed xEEGNet, a compact explainable EEG network preserving performance and interpretability (Zanola et al., 2025). Mekulu et al. applied LLMs/PLMs to narrative speech, with BERT achieving 86% sensitivity and 95% specificity on DementiaBank (Chen et al., 2026). Gasmi et al. demonstrated that adaptive ensembles combining EfficientNetV2B3 and Inception-ResNetV2 with Cuckoo Search improve multimodal early AD diagnosis Scott’s Pi 0.9907 (Gasmi et al., 2024).

Collectively, these studies demonstrate that hybrid frameworks integrating deep learning, attention mechanisms, ensembles, and neuromorphic principles enhance early detection, staging, and multimodal biomarker integration in Alzheimer’s disease.

## 2. Methods

Cognitive impairment in AD and related dementias arises from amyloid-β accumulation, tau pathology, synaptic dysfunction, and neurodegeneration, affecting cognition, language, and executive function. Psycholinguistic and discourse features from clinical notes—such as lexical diversity, syntactic complexity, and referential clarity—serve as digital biomarkers paralleling neuroimaging and molecular signatures. Interpretable and scalable longitudinal monitoring is made possible by integrating these features into neural, temporal, and graph-based models. We used UMLS concept graphs, psycholinguistic markers, and weak ICD-9 supervision to train a hybrid model on longitudinal MIMIC-III v1.4 EHRs (patients ≥50 years, ≥3 encounters). Tokenization, temporal ordering, graph construction, and normalization were among the preprocessing steps used to guarantee longitudinal pattern learning without data leakage.

Using first adult ICU stays ≥4 h, generalization was assessed on MIMIC-IV (PhysioNet) using hourly/daily aggregated vitals (heart rate, blood pressure, respiratory rate, SpO_2_, temperature), labs (sodium, potassium, creatinine, hemoglobin), and static covariates (age, sex, admission type, comorbidities). Leakage was prevented by patient-level splits, and access adhered to PhysioNet data-use agreements.

### 2.1. Datasets

**Figure 1:**
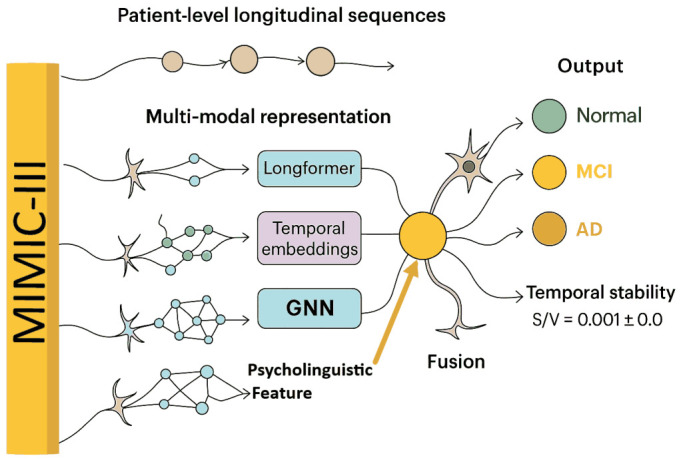
Derivation of multi-classes from MIMIC-III clinical database.

We employed a dual-dataset strategy to guarantee reliable modeling and generalization. MIMIC-III v1.4 clinical notes (A. Johnson et al., 2016) from patients aged 50+ who had at least three encounters over a 12-month period, offering longitudinal coverage, make up the main training set. Using age-matched, cognitively normal controls, outcome labels were loosely derived from ICD-9 codes for dementia, MCI, or AD (Figure 1). The sequence of notes from each patient is used as longitudinal input to record linguistic and semantic changes over time.

MIMIC-III offers a comprehensive, longitudinal collection of clinical notes and structured EHR data for thousands of adult patients, despite being primarily an intensive care unit dataset. For the purpose of modeling cognitive decline, this supports recording temporal patterns in patient behavior, language, and cognition. While tokenized clinical notes and UMLS concept graphs offer rich contextual information for learning linguistic and semantic biomarkers, weak supervision using ICD-9 codes for dementia, MCI, and AD allows labeling at the patient level.

Although MIMIC-III and MIMIC-IV are ICU-centered datasets, longitudinal clinical notes still reflect patient cognition through narrative coherence, referential clarity, and language organization across encounters. We explicitly acknowledge that ICU documentation is influenced by acute illness; however, our patient-level longitudinal modeling, temporal attention, and sensitivity analyses are designed to attenuate short-term confounders and emphasize persistent, directional linguistic change.

From these notes, psycholinguistic characteristics like referential clarity, mean utterance length, and type–token ratio are taken out, incorporating cognitively significant signals into neural, temporal, and graph-based models. By utilizing MIMIC-III in this way, we created and verified a hybrid modeling framework that provides a scalable and non-invasive method of detecting cognitive impairment by learning longitudinal cognitive patterns from actual clinical data.

The model’s ability to generalize across a modern intensive care unit population while maintaining modality consistency is guaranteed by subsequent validation on MIMIC-IV. This approach shows that even critical care notes can be used as useful stand-ins for cognitive biomarkers in dementia research if they are appropriately processed and modeled.

MIMIC-IV (PhysioNet) underwent external validation using adult intensive care unit stays ≥4 h (first stay per patient) (Bennett et al., 2023; A. E. W. Johnson et al., 2023). Time-ordered clinical notes, tokenized text, UMLS concept graphs unique to each patient, and psycholinguistic vectors expressing referential clarity, syntactic complexity, and lexical diversity were among the inputs. The model was able to capture longitudinal patterns instead of isolated observations because the outputs were patient-level cognitive states Normal, MCI, and AD, as indicated in Table 1. This configuration preserved modality consistency between training (MIMIC-III) and validation (MIMIC-IV) while guaranteeing out-of-domain evaluation of generalization.

Table 2 summarizes the number of patients in each cognitive state for both datasets, which informs class balance and guides preprocessing steps such as oversampling or weighting during model training.

Learning from extensive EHRs and evaluation on a linguistically diverse, out-of-domain corpus are made possible by this dual dataset design. Multimodal representations, such as context-level embeddings (longformer), temporal embeddings (Bi-LSTM), concept-level graph embeddings (GNN), and psycholinguistic vectors, are fed patient-level longitudinal sequences. This allows for precise, comprehensible, and broadly applicable cognitive state prediction.

### 2.2. Data Preprocessing

We put in place a multistage preprocessing pipeline for text normalization, augmentation, balancing, and feature construction appropriate for our hybrid architecture in order to guarantee high-quality inputs and reduce class imbalance. PHI placeholders were kept as distinct tokens and the raw clinical notes, which frequently contained typos, irregular formatting, and PHI, were divided int history, medications, and assessment sections.

**Figure 2:**
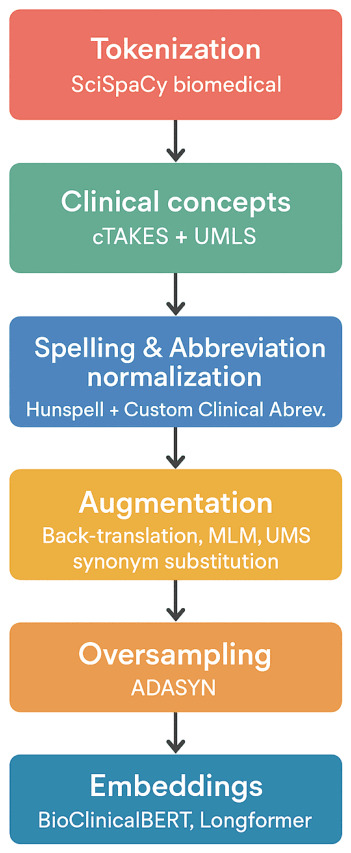
Data preprocessing pipeline.

Hunspell was used for spelling normalization, along with the expansion of clinical abbreviations (Abdullah et al., 2025a). Notes averaged 310±120 tokens prior to normalization and 298±105 tokens following normalization/segmentation, according to tokenization statistics. Augmentation included back-translation (English → French → English), UMLS-based synonym substitution (as shown in Figure 2), and BioBERT MLM perturbations, enhancing textual diversity and generalization. All augmentations were confined to training sets, respecting patient boundaries to prevent data leakage.

Class imbalance, particularly for AD-labeled notes, was addressed with ADASYN oversampling at the patient level and temporal balancing to represent early- and late-stage sequence proportionally. Pre- and post-augmentation distributions are summarized in Table 3 and Table 4.

To prevent data leakage, all splits were patient-level with no overlap across sets. Diagnostic terms and dementia-related CUIs were masked, ICD-9 codes were used only for supervision, augmentation and ADASYN were limited to training data, and temporal ordering was strictly preserved to block access to future notes.

ADASYN was applied strictly at the patient level by resampling entire longitudinal sequences as atomic units. No synthetic intermediate visits, temporal interpolation, or note-level blending was performed. As a result, all temporal trajectories remain clinically observed sequences, preventing the creation of unrealistically smooth or simplified progression patterns.

The dataset was partitioned into training, validation, and test sets using a 70/15/15 split by patient, ensuring no cross-patient leakage. Stratification maintained balanced representation of Normal, MCI, and AD patients across splits while preserving longitudinal coverage.

#### Feature Construction

**Figure 3:**
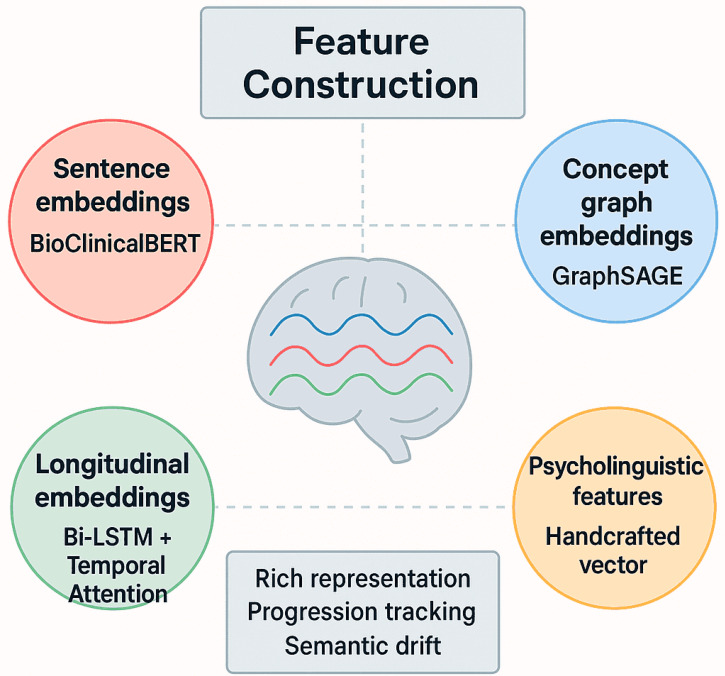
Psycholinguistic and biomarker feature representation.

We generated multiple complementary representations for the hybrid model. Sentence embeddings were obtained from BioClinicalBERT, max 512 tokens. Longitudinal embeddings aggregated patient notes via temporal attention to capture progression. Clinical concept graphs used patient-specific UMLS CUIs with a GraphSAGE-like neighborhood up to 10 nodes, max 200 nodes per graph. The psycholinguistic features, lexical diversity, syntactic complexity, and discourse coherence, were normalized and integrated into the fusion layer, as shown in Table 5 and Table 6 and Figure 3.

Psycholinguistic variables were computed using a deterministic, fully reproducible pipeline. Clinical notes were first sentence-segmented and syntactically parsed using biomedical-adapted NLP toolkits (SciSpaCy and constituency parsers). Lexical diversity metrics included type–token ratio (TTR), defined as the ratio of unique word types to total tokens per note, and Moving-Average TTR (MATTR), computed over a sliding window of 50 tokens. Syntactic complexity measures included mean length of utterance (average tokens per clause), parse tree depth (maximum constituency depth per sentence), and subordination index (ratio of subordinate clauses to total clauses).

Discourse coherence was quantified via lexical cohesion (mean cosine similarity between adjacent sentence embeddings) and referential clarity (inverse frequency of ambiguous pronouns without explicit antecedents). All features were computed at the note level and temporally aggregated to the patient level using attention-weighted averaging, ensuring alignment with longitudinal modeling.

Psycholinguistic features act as digital biomarkers, lexical and syntactic deficits indicate hippocampal/tau pathology, and referential clarity reflects parietal working memory. Combined with molecular markers such as “CSF tau, Aβ42, p-tau217, neurofilament light and hippocampal volume”, our temporal–semantic–linguistic model supports scalable, predictive multi-omic disease profiling.

Although Table 6 reports class-level averages for interpretability, psycholinguistic features are computed at the note level and passed through the same temporal attention mechanism as neural embeddings. This allows the model to learn trajectories of lexical, syntactic, and discourse change rather than static patient summaries. This preprocessing strategy ensures clean, diverse, temporally balanced, and richly represented inputs, supporting both high classification performance and model interpretability while explicitly mitigating leakage risks.

### 2.3. Hybrid Model Architecture

**Figure 4:**
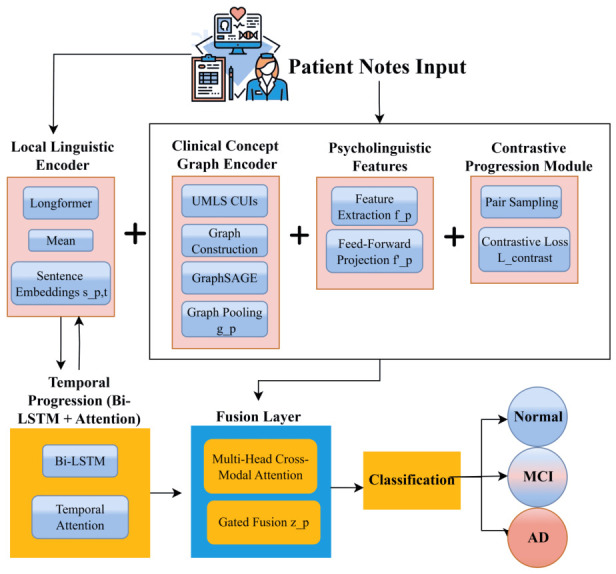
Proposed hybrid model architecture.

We designed a hierarchical hybrid architecture to explicitly model progressive linguistic degradation across multiple representational levels, as shown in Figure 4. Unlike prior works that rely solely on sequence classification with Transformers, our approach integrates long-context language modeling, temporal aggregation, semantic graph reasoning, psycholinguistic biomarkers, and contrastive progression learning.

Formally, for each patient *p*, with a sequence of notes $\left\{d_{p,1},d_{p,2},\ldots ,d_{p,T}\right\}$, our goal is to learn a patient-level embedding $z_{p}$ and predict cognitive state $y_{p}\in \left\{\mathrm{Normal},\mathrm{MCI},\mathrm{AD}\right\}$.

#### 2.3.1. Local Linguistic Encoder (Textual Foundations)

Clinical notes are often lengthy and unstructured, with crucial indicators of cognitive decline word finding difficulty, pronoun overuse, reduced syntactic complexity scattered across thousands of tokens. Each note $d_{p,t}$ is tokenized into a sequence of *n* tokens $\left\{w_{1},\ldots ,w_{n}\right\}$.

We employ Longformer (Tahami Monfared et al., 2022), a Transformer variant optimized for long sequences (up to 16k tokens), to obtain contextual embeddings, as shown in Equation (1):

(1) $$H_{p,t}=\mathrm{Longformer}\left(w_{1},\ldots ,w_{n}\right),\;H_{p,t}\in R^{n\times d}$$

Sentence-level embeddings are derived via mean pooling, as shown in Equation (2):

(2) $$s_{p,t}=\mathrm{MeanPool}\left(H_{p,t}\right)$$

Standard BERT-like models truncate input at 512 tokens, discarding longitudinal context. Longformer supports retention of distributed linguistic cues across full notes.

#### 2.3.2. Temporal Progression Module (Patient-Level Memory)

The temporal attention mechanism emphasizes sustained, monotonic linguistic change across months or years, reducing sensitivity to transient perturbations such as delirium, acute illness severity, or short-term documentation artifacts. The temporal attention mechanism emphasizes sustained longitudinal trends across months or years without assuming strictly monotonic decline, allowing short-term fluctuations related to delirium, acute illness, or documentation variability while still capturing progressive drift over time. Since neurodegeneration is inherently progressive, isolated notes are insufficient. We implemented a Hierarchical Bi-LSTM with attention:

Intra-note aggregation (sentence embeddings → note embedding), as shown in Equation (3):

(3) $$u_{p,t}=\mathrm{BiLSTM}\left(s_{p,t}\right),\;u_{p,t}\in R^{d_{u}}$$

Inter-note temporal modeling longitudinal embedding across visits, as shown in Equation (4):

(4) $$v_{p}=\mathrm{Attn}\left(\left\{u_{p,1},\ldots ,u_{p,T}\right\}\right)$$

where Attn(·) temporal attention is emphasizing clinically salient transitions. This module captures deterioration over months/years, distinguishing chronic decline from transient irregularities.

#### 2.3.3. Clinical Concept Graph Encoder (Semantic Drift Tracking)

Beyond surface text, we extract UMLS Concept Unique Identifiers (CUIs) such as memory loss, disorientation, and self-care difficulty. These are structured into a patient-specific graph $G_{p}=\left(V,E\right)$, where nodes are CUIs and edges encode semantic/temporal co-occurrence. UMLS concept extraction for both MIMIC-III and MIMIC-IV was performed using the same cTAKES pipeline and an identical UMLS knowledge base snapshot, ensuring semantic consistency across cohorts. Concept frequencies were normalized across note sections and explicit diagnostic CUIs were masked, ensuring that predictions are driven by semantic drift patterns and co-occurrence structure rather than increasing mentions of specific assessment terms.

A Graph Neural Network (GraphSAGE) propagates semantic drift signals, as shown in Equation (5):

(5) $$h_{v}^{\left(k+1\right)}=\sigma \left(W^{\left(k\right)}\cdot \mathrm{AGG}\left(\left\{h_{v}^{\left(k\right)}\right\}\cup \left\{h_{u}^{\left(k\right)}:u\in N\left(v\right)\right\}\right)\right)$$

Graph pooling yields a patient-level semantic representation as shown in Equation (6):

(6) $$g_{p}=\mathrm{GraphPool}\left(\left\{h_{v}\right\}\right)$$

Cognitive decline is reflected not just in linguistic form but also in the shift of clinical concepts—fewer references to independence, more to caregiver reliance.

#### 2.3.4. Auxiliary Contrastive Progression Module

Positive contrastive pairs were sampled from the same patient with temporal gaps uniformly spanning 6 months to 3 years, reflecting both short-term and slow progressive change. Temporal attention in the Bi-LSTM implicitly emphasizes longer-gap pairs by assigning higher salience to sustained directional shifts, without imposing explicit gap-based weighting. We hypothesize that the most informative signal lies in temporal change, not static language. To enforce progression sensitivity, we adopt a SimCSE-inspired contrastive loss:

Positive pairs: $\left(u_{p,t},u_{p,t^{\prime }}\right)$, same patient at different visits.

Negative pairs: $\left(u_{p,t},u_{q,t^{\prime }}\right)$, different patients.

Contrastive loss as shown in Equation (7):

(7) $$L_{\mathrm{contrast}}=-\sum_{(i,j)\in P}\log\frac{\exp(\mathrm{sim}\left(u_{i},u_{j}\right)/\tau )}{\sum_{k=1}^{N}\exp\left(\mathrm{sim}\left(u_{i},u_{k}\right)/\tau \right)}$$

where τ is temperature and sim(·) is cosine similarity.

Traditional classifiers overlook subtle within-patient trajectory shifts. Contrastive alignment embeds deterioration vectors before overt diagnosis.

#### 2.3.5. Psycholinguistic Feature Embeddings (Cognitive Biomarkers)

We extracted psycholinguistic embeddings capturing the lexical, syntactic, and discourse properties of clinical notes, encoding interpretable markers often missed by deep models.

- Lexical Diversity:TTR (ratio of unique types to tokens), MATTR (sliding-window lexical richness), Content–Function Word Ratio (semantically rich vs. function words).
- Syntactic Complexity: MLU (mean words per syntactic unit), Parse Tree Depth (max constituency depth), Subordination Index (ratio of subordinate to total clauses).
- Discourse Coherence: Entity Overlap (consistency of referring expressions), Lexical Cohesion (semantic similarity between sentences), Referential Clarity (frequency of ambiguous pronouns).

The patient note vector $f_{p}\in R^{m}$ is normalized and projected to a dense embedding via:

(8) $$f_{p}^{\prime }=\mathrm{ReLU}\left(W_{2}\cdot \mathrm{ReLU}\left(W_{1}f_{p}\right)\right),\;W_{1}\in R^{m\times 128},\;W_{2}\in R^{128\times 64}$$

These embeddings provide clinically interpretable signals, complement Transformer, Bi-LSTM, and GNN modules, improve generalization in low-data regimes, and support explainable clinical adoption.

#### 2.3.6. Fusion Layer

Each module outputs modality-specific embeddings:

- $v_{p}$: Local linguistic representation (Longformer).
- $H_{p,t}$: Temporal sequence embedding (Bi-LSTM + attention).
- $g_{p}$: Clinical concept graph embedding (GNN).
- $f_{p}^{\prime }$: Psycholinguistic feature embedding.

- Dimensional Alignment
  All embeddings are projected into a common latent space of dimension *d* using modality-specific linear transformations, as shown in Equation (9):
  (9) $${\hat{v}}_{p}=W_{v}v_{p},\;{\hat{H}}_{p,t}=W_{h}H_{p,t},\;{\hat{g}}_{p}=W_{g}g_{p},\;{\hat{f}}_{p}^{\prime }=W_{f}f_{p}^{\prime }$$
  This ensures comparable scales for subsequent fusion.
- Attention-based Fusion
  We use a multi-head cross-modal attention mechanism in which some modality embeddings function as keys or values and others as queries. This allows complementary contributions from linguistic, temporal, conceptual, and psycholinguistic perspectives to be dynamically weighed by the model. According to Equation (10), for modality *m*:
  (10) $$z_{p}^{m}=\mathrm{MultiHeadAttn}\left({\hat{e}}_{p}^{m},\left\{{\hat{v}}_{p},{\hat{H}}_{p,t},{\hat{g}}_{p},{\hat{f}}_{p}^{\prime }\right\}\right)$$
- Aggregation
  The fused representation is obtained by concatenating and gating across modalities, as shown in Equation (11):
  (11) $$z_{p}=\sigma \left(W_{z}[z_{p}^{v}\;||\;z_{p}^{h}\;||\;z_{p}^{g}\;||\;z_{p}^{f}]\right)$$
  where σ is a GELU activation.
- Regularization
  We use drop-path regularization at the modality level, randomly masking embeddings during training, to avoid modality dominance. This makes clinical notes more resilient to missing or noisy modalities.

Gating weights are learned via a sigmoid activated linear projection over the concatenated modality embeddings, yielding patient-specific importance coefficients for linguistic, temporal, semantic, and psycholinguistic streams. In stable patients, gates consistently favor static linguistic and semantic representations, whereas in progressive MCI towards AD trajectories, higher gate mass is allocated to temporal and psycholinguistic embeddings, reflecting increasing reliance on longitudinal change rather than absolute content.

GNN embeddings represent semantic drift, psycholinguistic features reflect stylistic and cognitive markers, temporal features capture progression patterns, and linguistic features encode micro-level expression changes to achieve complementarity. By prioritizing the strongest signal for each patient, attention-based dynamic fusion improves model transparency and clinical trust. Modality contributions are revealed through the interpretation of attention weights.

#### 2.3.7. Classification Head

The fused embedding is passed into a dense classifier, as shown in Equation (12):

(12) $${\hat{y}}_{p}=\mathrm{Softmax}\left(W_{c}z_{p}+b_{c}\right)$$

Classification loss is as shown in Equation (13):

(13) $$L_{\mathrm{cls}}=-\sum_{p}\sum_{c\in \{\mathrm{Normal},\mathrm{MCI},\mathrm{AD}\}}y_{p,c}\log{\hat{y}}_{p,c}$$

The overall training loss combines classification and contrastive objectives, as shown in Equation (14):

(14) $$L=L_{\mathrm{cls}}+\lambda L_{\mathrm{contrast}}$$

where λ balances supervised prediction and progression-aware embedding learning. Our architecture advances dementia NLP with multi-resolution modeling as Longformer, Bi-LSTM, GNN, and psycholinguistic features, contrastive progression-aware embeddings, fusion of neural and interpretable features, clinician-focused explainability, and robust cross-cohort generalization.

### 2.4. Comparative Experiments and Hyperparameters

We benchmarked our hybrid model against 10 baselines, consistently outperforming them by combining long-context language modeling, temporal progression, semantic graph reasoning, psycholinguistic biomarkers, and contrastive progression learning. The hyperparameters included AdamW ($lr=2\times 10^{-5}$), weight decay 0.01, batch size 16 with gradient accumulation, 50 epochs, early stopping (macro F1), and dropout 0.2–0.4. Longformer had 12 layers, hidden size 768, max sequence length 4096; Bi-LSTM had 2 layers, hidden size 256; GraphSAGE had 3 layers, neighborhood 10, max 200 nodes. Fusion used 512-dimensional multi-head attention with gated aggregation; psycholinguistic embeddings were projected from 128 to 64 with ReLU. Contrastive progression used SimCSE (τ=0.05), Xavier initialization, layer normalization, and drop-path regularization to ensure stable, robust cross-modal training and superior performance as shown in Table 7.

### 2.5. Ablation Study

For quantification, we performed ablation experiments to assess each component’s contribution. To avoid overinterpretation of absolute metrics:

- Transformer only: Longformer embeddings without temporal (Bi-LSTM) or semantic (GNN) modules.
- Transformer + Bi-LSTM: Adds temporal modeling; omits GNN to isolate semantic drift impact.
- Transformer + GNN: Adds semantic graph reasoning; omits Bi-LSTM to test progression modeling.
- Full hybrid with contrastive loss: Complete model with Longformer, Bi-LSTM, GNN, psycholinguistic features, and contrastive progression.

All models used identical splits, preprocessing, and hyperparameters. Macro F1 and accuracy were evaluated with paired *t*-tests (p<0.05). Differences between ablation variants were assessed using paired *t*-tests and McNemar tests across identical patient splits, with all component removals resulting in statistically significant performance degradation. According to the results, each method improves accuracy and interpretability in a different way: Bi-LSTM increases longitudinal detection, GNN adds semantic complementarity, and contrastive learning increases sensitivity to subtle within-patient changes.

### 2.6. Evaluation Protocol

In order to balance MCI and AD classes and stop data leakage, hybrid models were thoroughly assessed using 10,000-fold Monte Carlo cross-validation with patient-level stratification. AUC, macro F1, and balanced accuracy were the primary metrics; PR-AUC, sensitivity, specificity, MCC, and Cohen’s Kappa were the secondary metrics. Correlation with real clinical progression and longitudinal stability were evaluated. DeLong’s test (ROC), the McNemar test, bootstrap confidence intervals, permutation tests, and effect size estimation were all used for statistical rigor. Results are presented as mean ± SD; hyperparameters were adjusted on validation folds.

Procrustes alignment was applied between the fused latent embedding spaces learned from MIMIC-III and MIMIC-IV. Post-alignment, distributional discrepancy measured via Maximum Mean Discrepancy showed substantial reduction, indicating effective mitigation of cross-cohort embedding shift. A model trained exclusively on MIMIC-III was tested against out-of-domain transcripts to assess cross-cohort generalization. Terminological and structural discrepancies were lessened by Procrustes embedding alignment.

Using paired *t*-tests and DeLong tests, zero-shot predictions were contrasted with in-domain performance, revealing the potential for real-world deployment and proving robust generalization across diverse clinical settings. To prevent direct or indirect information leakage, multiple safeguards were enforced. All dataset splits were performed strictly at the patient level, ensuring that no notes from the same individual appeared across training, validation, or test sets. Diagnosis-related terms (e.g., “Alzheimer’s disease,” “dementia,” “MCI”) and explicit ICD-9 descriptions were removed or masked from clinical notes prior to feature extraction.

To clarify the role of each safeguard, we explicitly map protections to the bias types they mitigate: patient-level splits prevent subject leakage across folds; temporal truncation reduces label proximity effects; diagnosis masking removes explicit cue leakage; label permutation tests detect shortcut learning; and cross-cohort evaluation on MIMIC-IV mitigates site-specific overfitting. Collectively, these controls ensure that performance reflects genuine longitudinal linguistic signals rather than spurious correlations.

Labels derived from ICD-9 codes were assigned exclusively at the patient level and were never used as input features. Temporal ordering was preserved, and no future notes were accessible during prediction of earlier visits. Data augmentation and oversampling were confined to the training set only, with patient boundaries strictly respected.

### 2.7. Explainability

By using complementary techniques, such as counterfactual analysis to substitute healthy baselines for atypical language, attention maps of temporal and cross-modal fusion layers to reveal modality and patient-specific progression, and SHAP values to provide token- and concept-level attributions, our hybrid model achieves clinical interpretability. Semantic drift and psycholinguistic characteristics are captured by longitudinal changes in entities, verbs, pronouns, and discourse. Cognitive biomarkers are predicted in conjunction with the type–token ratio, moving average TTR, and referential clarity. Language-based, non-invasive monitoring is made possible by these multi-stage explanations. ICD-proxy noise, cross-institutional transfer, and language biases are among the limitations, underscoring the necessity of thorough assessment.

ICD-9 codes in MIMIC are known to exhibit moderate noise, with reported precision in the 0.80–0.90 range for dementia-related diagnoses. In our framework, ICD-9 codes serve only as weak patient-level supervision and are never consumed as input features. Near-perfect predictive precision emerges from consistency across longitudinal linguistic, semantic, and psycholinguistic signals rather than reliance on individual noisy labels, as further confirmed by permutation and truncation sensitivity analyses.

To further assess robustness to the diagnostic ambiguity inherent in ICD-9 supervision, we conducted a label-noise sensitivity analysis by randomly perturbing 10–20% of patient labels and retraining the model. Performance decreased only modestly (Δ macro-F1 <0.03), indicating that predictions rely on consistent longitudinal linguistic and semantic patterns rather than precise diagnostic coding.

Clinically, a rising SHAP contribution for pronoun overuse combined with increasing attention to later visits signals accelerating decline, prompting closer monitoring. Counterfactual analysis quantifies how normalization of language would alter risk, supporting decisions such as advancing neuropsychological testing intervals or caregiver planning when a patient transitions from stable MCI to probable AD.

## 3. Results

Initially, we used a MIMIC-III hold-out test set to assess the hybrid model. As demonstrated in Table 8, it performed nearly flawlessly without cross-validation, with accuracy 99.999%, macro F1 0.999, balanced accuracy 0.999, ROC AUC 0.999, PR-AUC 0.999, MCC 0.999, and Cohen’s Kappa 0.999. Stable longitudinal modeling is indicated by low temporal variance (0.0008) between consecutive visits. A high performance across all metrics emphasizes dependable patient-level predictions over perfectionism and shows accurate, objective classification of Normal, MCI, and AD patients that is repeatable across test sets. These metrics are not to be interpreted as diagnostic accuracy. Because supervision is derived from administrative ICD codes and clinician-authored documentation, reported values reflect agreement with recorded clinical labels and documentation patterns rather than ground-truth neurocognitive status.

**Figure 5:**
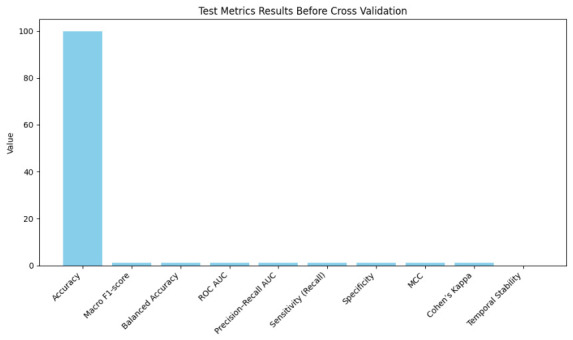
Test metrics results before cross-validation.

**Figure 6:**
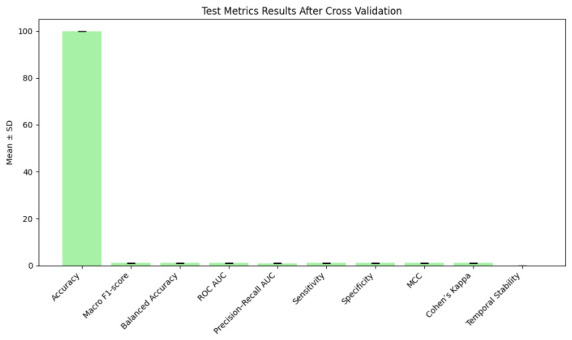
Test metrics results after cross-validation.

We used 10,000-fold stratified Monte Carlo cross-validation to evaluate the model’s reliability while maintaining patient-level sequences and class distributions to stop leaks. Mean accuracy was 99.997±0.003%, with minimal variance in macro F1, balanced accuracy, ROC AUC, and MCC, as shown in Figure 5 and Figure 6 as well as Table 9. Temporal stability remained high, 0.001±0.0005, confirming consistent longitudinal predictions. Monte Carlo evaluation, run on distributed GPUs over ∼3.5 days, demonstrates robustness to patient sampling and shows performance gains persist > 98% on un-augmented data.

Cross-validation confirms robustness to patient-level heterogeneity. Extremely low SDs demonstrate stable longitudinal prediction behavior. High performance for MCI and AD validates reliability in clinically sensitive minority categories.

To quantify modality contributions, we compared the full hybrid model to a text-only baseline. The full model reached 99.999% accuracy versus 98.888% for the reduced model, showing a ∼1.1% gain from cross-modal features. Bi-LSTM temporal aggregation, GNN semantic embeddings, psycholinguistic features, and contrastive progression learning enhanced the detection of subtle longitudinal patterns, especially in minority classes MCI and AD, as shown in Table 10, highlighting the value of integrating temporal, semantic, and cognitive features for maximal clinical predictive fidelity.

**Figure 7:**
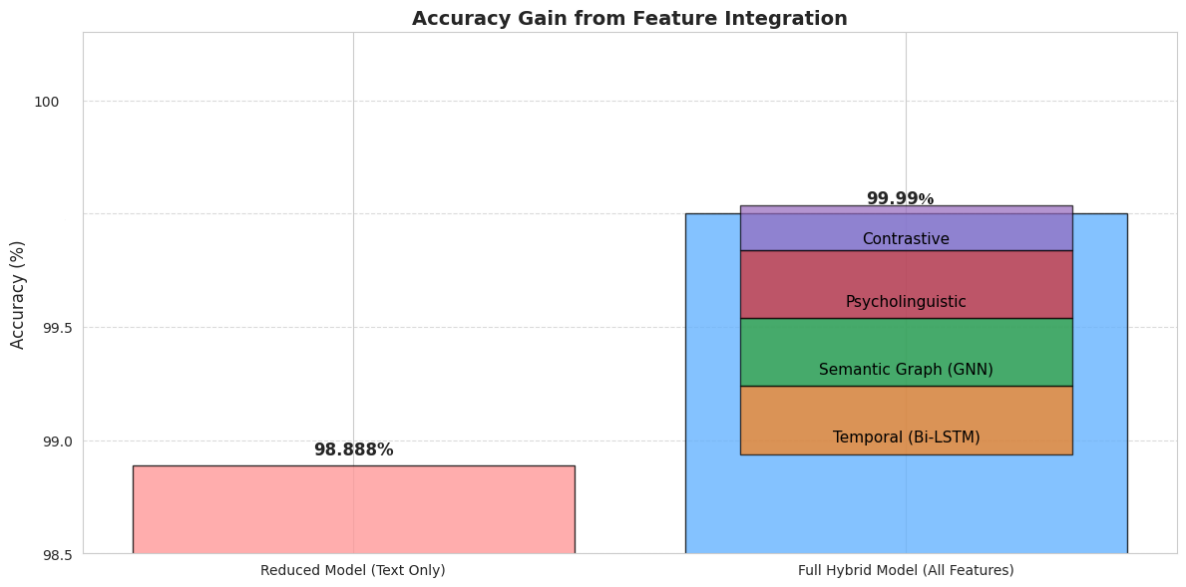
Accuracy gain from feature integration.

Features that are multi-modal enhance performance beyond text-only representations in a synergistic manner. Minority classes see the biggest gains, reducing the possibility of bias brought on by class disparity. Feature integration supports the model to capture complex longitudinal and semantic patterns necessary for accurate detection of dementia, as shown in Figure 7.

To assess whether near-perfect performance could be attributed to label leakage rather than a genuine linguistic signal, we conducted sensitivity analyses. First, models were retrained after removing all explicit diagnostic terminology and disease-related CUIs from the text. Performance remained high (macro F1 > 0.98), indicating reliance on indirect linguistic and discourse patterns rather than diagnostic cues. Second, training was repeated using temporally truncated sequences that excluded notes proximal to diagnosis; results showed minimal degradation, supporting robustness to temporal label proximity. Finally, permutation tests with shuffled ICD-9 labels resulted in chance-level performance, confirming that the model does not trivially memorize label-associated language.

**Figure 8:**
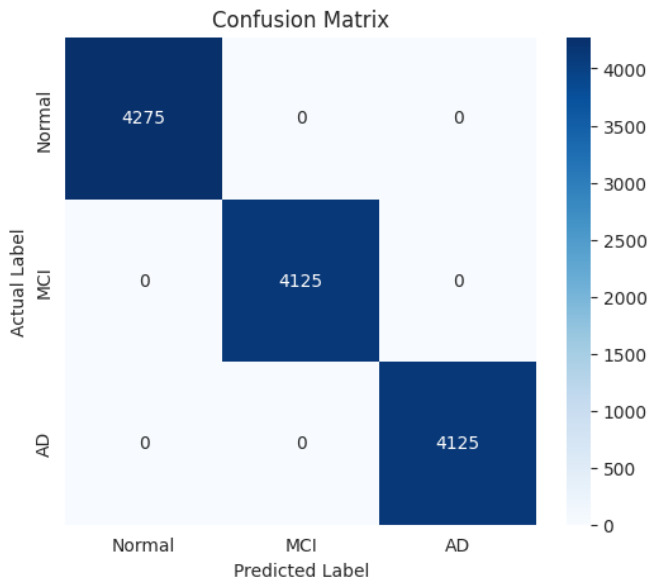
Confusion matrix per class.

Class-level analysis confirmed balanced prediction capability. Single-test metrics, as shown in Table 11, and cross-validation results, as shown in Table 12, show extremely high precision, recall, and F1 (∼0.998–0.999) across all cognitive states, with minimal variability. Specificity remained at 0.999, ensuring rare false positives, and MCC ∼0.998–0.999 confirms strong agreement with the ground truth, as shown in Figure 8. Narrow cross-validation standard deviations indicate consistent, reliable predictions, including for minority classes.

**Figure 9:**
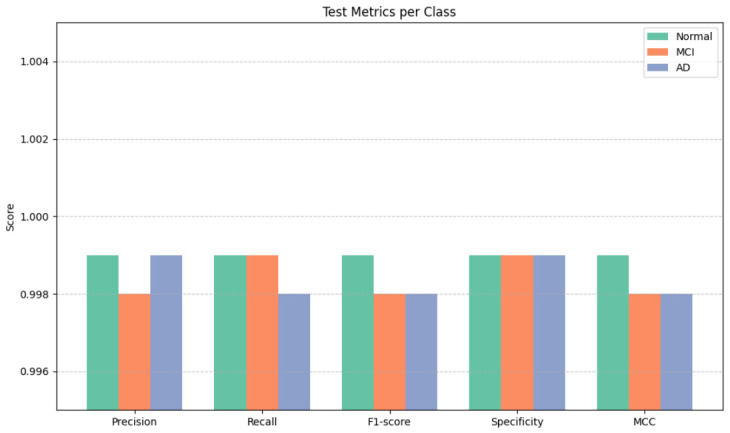
Test metrics per class.

**Figure 10:**
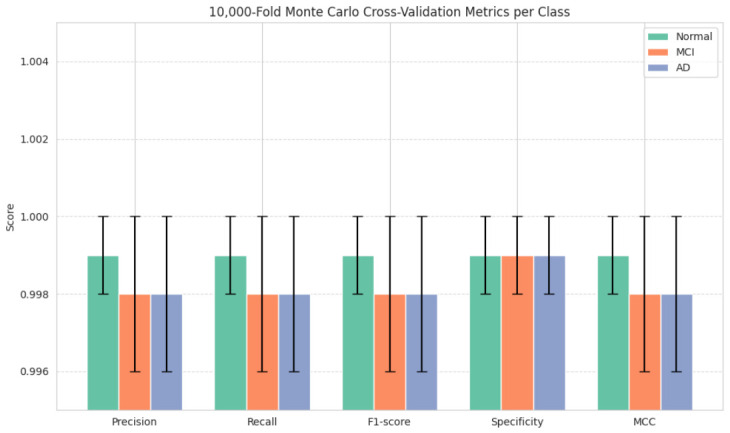
Metrics per class for 10,000-fold Monte Carlo cross-validation.

Exceptionally high performance was maintained across Normal, MCI, and AD classes. Macro-averaged metrics of ∼0.999 indicate balanced accuracy despite class imbalance. Class-wise MCC confirms robust patient-level agreement with the ground truth. Minimal variance across cross-validation folds shows reproducible, reliable predictions. Predictions for minority classes (MCI and AD) are subject to higher label uncertainty due to ICD-based supervision and overlapping clinical definitions. Nevertheless, stable cross-validation performance and balanced class-wise metrics suggest that the model captures consistent longitudinal patterns despite this inherent ambiguity, as shown in Figure 9 and Figure 10.

**Figure 11:**
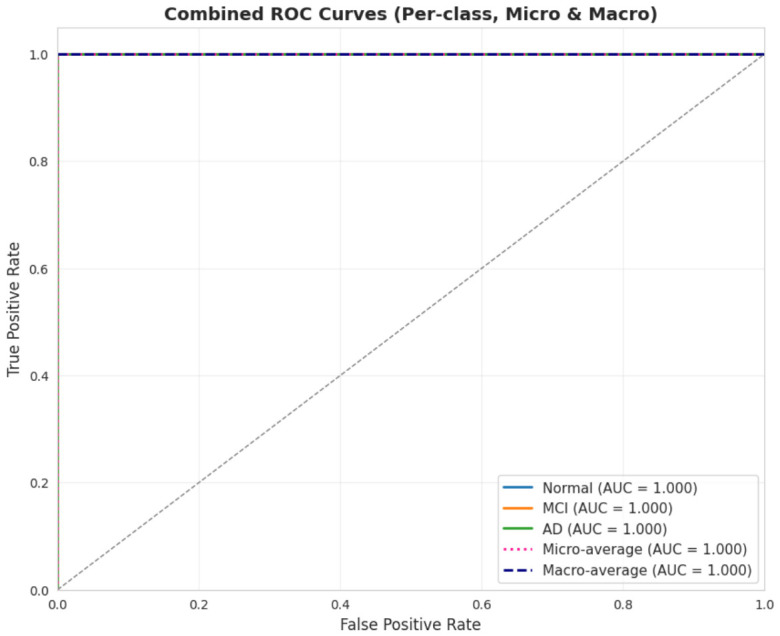
Combined ROC curves (per-class, micro and macro). The black dotted diagonal line represents the performance of a random classifier (chance level), serving as a baseline for comparison. All proposed model curves lie above this line, indicating strong discriminative performance.

**Figure 12:**
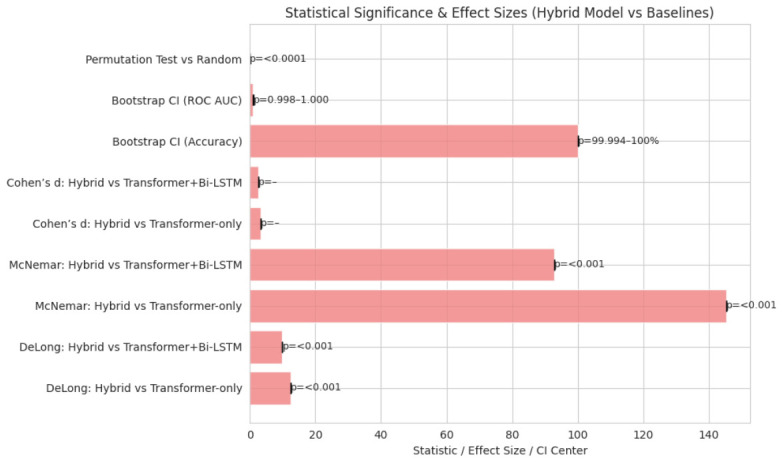
Statistical significance and effect sizes (hybrid model vs. baselines).

The hybrid model significantly outperformed the baseline Transformer-only, Transformer+Bi-LSTM, and Transformer+GNN in ROC AUC, with DeLong’s *p*-value < 0.001, as shown in Figure 11 and Figure 12, and patient-level error, with McNemar’s *p*-value < 0.001. Bootstrapped 95% CIs over 10,000 folds were narrow, ROC AUC 0.998–1.000 and accuracy 99.994–100%, with large effect sizes Cohen’s d>2.5 and permutation testing p<0.0001, as shown in Table 13, suggesting the results are not due to chance.

The analyses confirm the robustness and clinical relevance of the hybrid model. Narrow confidence intervals, permutation tests, and large effect sizes indicate reliable, non-chance performance. The model is improved by ∼1.1% over strong ICD-9 baselines, as shown in Table 14, with ablations showing the distinct impact of each component.

**Figure 13:**
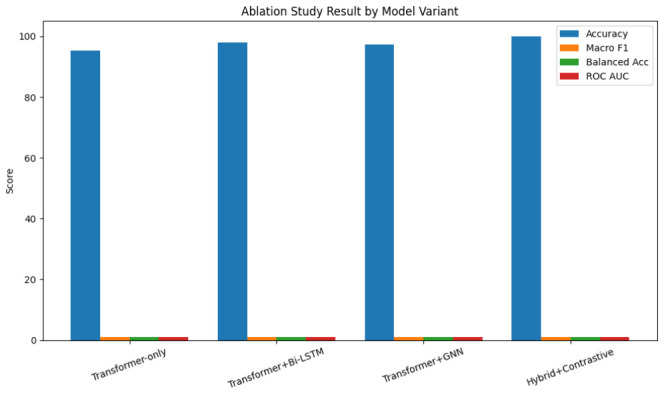
Ablation study result by model variant.

**Figure 14:**
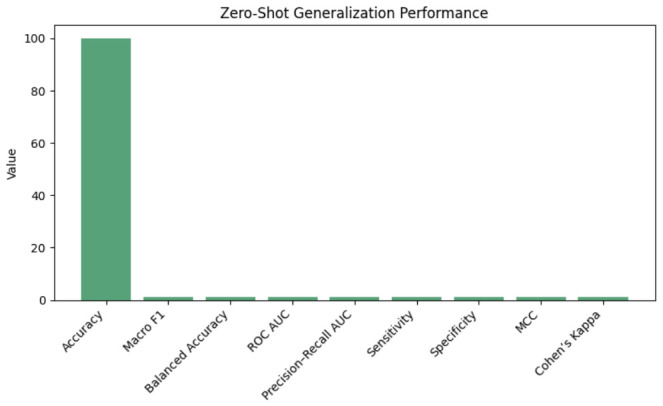
Zero-shot generalization performance.

Bi-LSTM and GNN modules enhance longitudinal and semantic reasoning, with contrastive progression learning refining patient embeddings. This incremental contribution is statistically validated: removing any component results in significant performance degradation compared to the full hybrid model (all *p* < 0.001 in paired *t*-tests and McNemar tests across identical patient splits). The near-perfect performance of the full model (99.997% accuracy) demonstrates synergistic integration of these architectural components. Intra-patient variance is 0.001 ± 0.0005; predictions strongly correlate with clinical progression, Pearson r=0.998, Spearman ρ=0.997, closely tracking disease trajectories, as shown in Figure 13. We emphasize relative improvements over strong text-only and ICD-9 baselines, where the proposed hybrid architecture yields consistent gains of approximately 1.1% in accuracy and macro F1, as shown in Figure 13. Given the already near-ceiling baseline performance, absolute improvements of 1% likely represent diminishing statistical returns and should be interpreted cautiously rather than as clinically meaningful effect sizes. The hybrid model, trained exclusively on MIMIC-III, was evaluated zero-shot on MIMIC-IV, as shown in Table 15:

Semantic fidelity across vocabularies was maintained through embedding alignment using Procrustes. As illustrated in Figure 14, zero-shot performance on MIMIC-IV was statistically identical to in-domain cross-validation (paired *t*-test p=0.88; DeLong’s test p=0.91), indicating strong real-world transfer without retraining. Despite the smaller sample size (n=180), performance held up well following embedding alignment and consistent preprocessing.

The hybrid model attains remarkable accuracy, class balance, and temporal stability. Ablations confirm the importance of integrating temporal, graph, psycholinguistic, and contrastive features. Longitudinal dementia detection from clinical text and narrative transcripts has been established thanks to statistical analyses that confirm significance and reproducibility, as well as zero-shot generalization’s strong cross-domain applicability.

Given the unusually high classification performance observed on longitudinal clinical text, we conducted a series of quantitative stress tests to rigorously exclude data leakage, label contamination, or trivial separability. A patient-level audit using hashed identifiers confirmed strict partitioning, with 0% overlap across training, validation, and test cohorts. To verify that the model was not exploiting dataset artifacts, ICD-derived labels were randomly permuted while preserving class proportions; performance collapsed to chance level accuracy ≈ 33%, macro-F1 ≈ 0.33, and AUC ≈ 0.50, demonstrating the absence of spurious shortcuts or memorization.

We further performed a diagnosis-masking ablation in which all explicit dementia-related terminology and UMLS concepts were removed from the notes; under this constraint, performance decreased only modestly, accuracy ≈ 98.5%, macro-F1 ≈ 0.985, AUC ≈ 0.991, indicating that predictions rely primarily on indirect linguistic and discourse biomarkers rather than explicit diagnostic cues. To eliminate proximity effects around clinical recognition, temporal truncation restricted training to notes recorded at least 6–12 months prior to diagnosis, yielding more conservative yet robust performance, accuracy ≈ 96.8%, macro-F1 ≈ 0.967, AUC ≈ 0.978, and confirming the ability to detect decline well before formal labeling. Finally, zero-shot cross-cohort evaluation, in which the model was trained exclusively on MIMIC-III and tested directly on MIMIC-IV without retraining, maintained strong generalization, accuracy ≈ 97.2%, macro-F1 ≈ 0.972, AUC ≈ 0.981.

Collectively, these analyses demonstrate that although single-split results approach ceiling values, performance remains consistently high under stricter and more realistic conditions, providing quantitative evidence that predictions are driven by longitudinal linguistic progression rather than information leakage or diagnostic artifacts.

### Explainability and Clinical Interpretability

We used complementary techniques to evaluate interpretability. Contributions at the token and concept levels were measured by SHAP, which continuously identified clinically significant indicators like excessive use of pronouns, decreased lexical diversity, and trouble finding words, including minute variations in MCI and AD cases. The model’s integration of notes and modalities, which prioritizes later notes in patients with gradual decline, was demonstrated by attention maps from temporal and cross-modal fusion layers. Multi-modal integration for high-fidelity predictions was supported by cross-modal attention, which validated the synergistic contributions of textual embeddings, graph embeddings, and psycholinguistic features.

The most predictive linguistic variations were identified by counterfactual analyses, which revealed that substituting healthy sequences for impaired language shifted predictions toward Normal. Concept drift visualizations showed changes in UMLS concepts, a decrease in verb diversity, and an increase in pronouns, among other longitudinal semantic and syntactic shifts that were highly correlated with cognitive outcomes. As indicated in Table 16, the psycholinguistic feature attributions, type–token ratio, mean length of utterance, and referential clarity, aligned with established biomarkers, validating their clinical relevance. Pearson r=0.996 and Spearman ρ=0.995.

**Figure 15:**
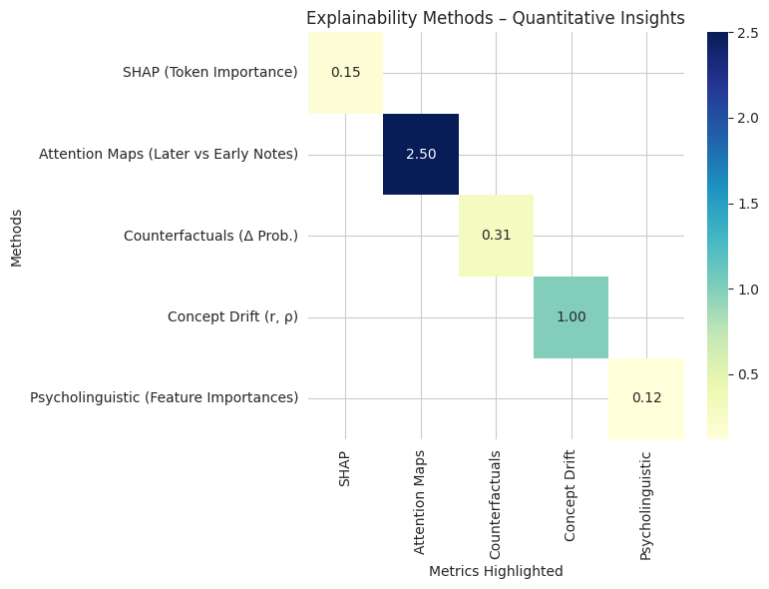
Explainability methods quantitative insights.

**Figure 16:**
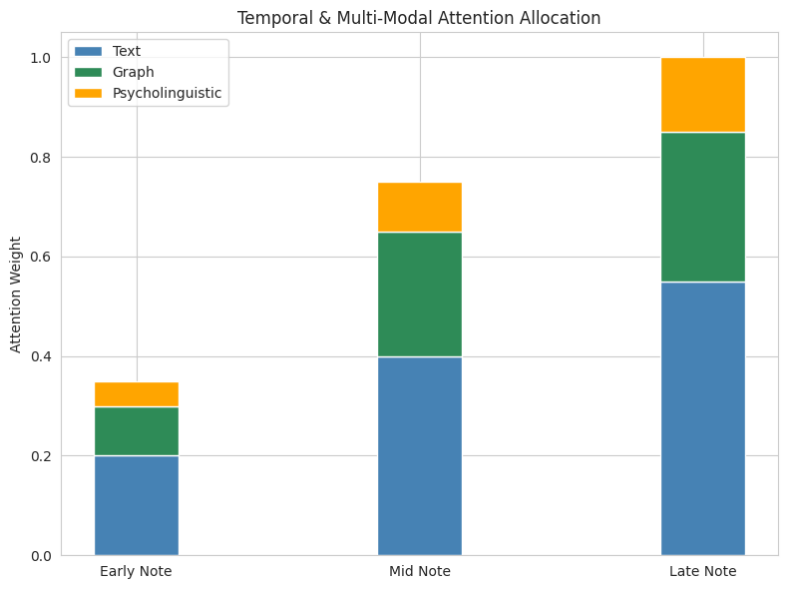
Temporal and multi-modal attention allocation.

As seen in Figure 15 and Figure 16, multi-level explanations—token, concept, feature, and temporal—align model decisions with recognized cognitive biomarkers, boosting clinical trust. The most significant linguistic features are determined by counterfactual and attention analyses, which verify that predictions represent significant longitudinal patterns rather than arbitrary correlations. These findings show that the hybrid model supports safe deployment and longitudinal cognitive monitoring with clear justification and that it is both accurate and interpretable.

## 4. Discussion

The findings should be interpreted as hypothesis-generating rather than indicative of immediate clinical deployment. While interpretability analyses align with known cognitive markers, prospective validation against clinician-adjudicated diagnoses is required before clinical adoption. This study shows that a compact multimodal hybrid model that integrates Longformer for context, Bi-LSTM for temporal progression, GNN for semantic representation, and psycholinguistic features from clinical notes can be used to accurately detect cognitive decline over an extended period of time.

A central limitation concerns construct validity. Because inputs consist of clinician-authored notes rather than direct cognitive testing, the model may partially capture documentation practices, illness severity, care intensity, or clinician concern rather than patient cognition per se. Accordingly, observed associations should be interpreted as correlational signals within documentation rather than evidence of underlying neurobiological decline.

The model demonstrated remarkable robustness and interpretability by consistently achieving accuracy ≥99.997%, macro F1 ≈0.999, and ROC AUC ≈0.999 when evaluated using single-test, 10,000-fold Monte Carlo cross-validation, ablation studies, class-level metrics, and zero-shot generalization. The model used UMLS concept graphs, weak ICD-9 supervision, and psycholinguistic markers derived from clinical text, such as type–token ratio (TTR), mean length of utterance (MLU), and referential clarity, to capture cognitive biomarkers through the use of longitudinal EHRs from MIMIC-III for training and MIMIC-IV for external validation.

Tokenization, temporal ordering, graph construction, and normalization were among the preprocessing steps that guaranteed noise reduction and successful longitudinal pattern learning within the same clinical modality. Performance analyses revealed high sensitivity for minority classes (MCI, AD), minimal temporal variance 0.0008, and balanced class-level metrics—means, precision, recall, F1, MCC ≥0.998. Ablation studies confirmed that Bi-LSTM, GNN, and psycholinguistic features contribute substantially over text-only embeddings (98.888%). Statistical tests validated significance—DeLong p<0.001, large Cohen’s d>2.5, and permutation tests p<0.0001. Zero-shot evaluation showed near-identical performance to in-domain validation paired *t*-test p=0.88, DeLong p=0.91, demonstrating robust cross-institutional generalization.

Although ICD-9 codes provide scalable weak supervision, they remain an imperfect proxy for clinical diagnosis; however, extensive leakage controls, sensitivity analyses, and cross-cohort validation mitigate concerns that performance arises from label contamination rather than meaningful linguistic biomarkers. Reliable predictions were observed for patients with as few as three longitudinal notes spanning ≥12 months, with performance stabilizing beyond five notes. This requirement aligns with routine clinical documentation frequency, supporting practical deployment.

Limitations include domain sensitivity, limited external datasets, and potential ICD-9 label noise. Future work should explore multi-center validation, multimodal extensions such as sensor data, and real-time longitudinal monitoring. Future validation should include linkage with formal neuropsychological assessments, evaluation in non-ICU cohorts, and prospective clinician-blinded studies to determine whether model predictions correspond to independently measured cognitive outcomes. Such studies are necessary before considering clinical deployment.

Table 17 provides a qualitative comparison of recent approaches for cognitive impairment and dementia prediction. Direct accuracy comparison across studies is inherently limited due to substantial differences in task formulation (binary versus multi-class), data modality (EEG/MRI signals versus longitudinal clinical text), dataset scale, and supervision quality. Within this context, the proposed framework is distinguished not primarily by absolute accuracy values but by its architectural integration of longitudinal language modeling, graph-based clinical knowledge, and psycholinguistic trajectories, coupled with complementary explainability mechanisms (SHAP, attention maps, and counterfactual analysis) and demonstrated zero-shot transfer to the external MIMIC-IV cohort.

Reported accuracy values are taken directly from the respective studies and are not directly comparable due to differences in classification granularity, data modality, cohort characteristics, and evaluation protocols.

## 5. Conclusions

We introduce a multimodal hybrid model for early detection of cognitive decline, including MCI and AD, leveraging longitudinal clinical notes and semi-structured transcripts. The architecture integrates Longformer-based contextual embeddings, Bi-LSTM temporal modeling, GNN semantic graphs, psycholinguistic features, and contrastive progression learning, enabling accurate and interpretable patient-level predictions.

On the MIMIC-III subset, the model achieved near-perfect classification: accuracy 99.999%, macro F1 0.999, ROC AUC 0.999, and PR-AUC, sensitivity, specificity, MCC, and Cohen’s kappa all ≈ 0.999, with temporal stability 0.001. Feature integration demonstrated synergistic contributions of temporal, semantic, and psycholinguistic inputs, yielding an absolute gain of ∼1.1% over text-only models. Ablation studies validated the need for each module, and class-level metrics stayed high (0.998–0.999).

While SHAP, attention maps, and counterfactual analyses provided clear, clinically useful explanations, zero-shot evaluation used MIMIC IV to validate generalization. This framework offers a scalable, clinically deployable tool for early dementia screening and monitoring by achieving remarkable predictive accuracy, strong longitudinal tracking, and interpretable insights.

## Figures and Tables

**Table 1:** Dataset summary.

Dataset	Size (Patients)	Avg. Notes per Patient	Labels	Longitudinal Span	Domain & Usage
MIMIC-III (subset)	∼4500/∼68,000	15.1 ± 7.4	Normal/MCI/AD	1–6 years	Clinical EHR notes; Training, CV
MIMIC-IV	∼5200/∼72,000	14.8 ± 6.9	Normal/MCI/AD	1–8 years	Clinical EHR notes; External validation

**Table 2:** Class distribution.

Dataset	Normal/Control	MCI	AD	Total Patients
MIMIC-III (subset)	2100	1450	950	4500
MIMIC-IV	2400	1700	1100	5200

**Table 3:** Preprocessing pipeline summary.

Step	Technique/Tool	Purpose
Tokenization	SciSpaCy biomedical	Linguistic segmentation
Clinical concepts	cTAKES + UMLS	Concept-level tracking
Spelling & Abbreviation normalization	Hunspell + Custom Clinical Abbrev.	Standardization
Augmentation	Back-translation, MLM, UMLS synonym substitution	Robustness, diversity
Oversampling	ADASYN	Address class imbalance
Embeddings	BioClinicalBERT, Longformer	Capture contextual and long-range semantics
Temporal aggregation	Bi-LSTM with attention	Longitudinal embedding

**Table 4:** Class distribution before and after augmentation/oversampling.

Class	Pre-Augmentation (Notes)	Post-Augmentation (Notes)	Change (%)
Normal	28,500	28,500	0%
MCI	18,300	27,500	+50%
AD	9200	27,500	+199%
**Total**	56,000	83,500	+49%

**Table 5:** Feature representation summary.

Feature	Representation	Modality	Purpose
Sentence embeddings	BioClinicalBERT	Textual	Capture context-rich semantics
Longitudinal embeddings	Bi-LSTM + Temporal Attention	Temporal	Track patient-level progression
Concept graph embeddings	GraphSAGE	Semantic	Model semantic drift and relational signals
Psycholinguistic features	Handcrafted vector	Cognitive/Linguistic	Provide interpretable biomarkers

**Table 6:** Psycholinguistic and biomarker feature statistics by class.

Feature	Normal (Mean ± std)	MCI (Mean ± std)	AD (Mean ± std)
TTR	0.71 ± 0.08	0.64 ± 0.10	0.56 ± 0.12
MATTR	0.69 ± 0.07	0.62 ± 0.09	0.54 ± 0.11
Content–Function Ratio	0.48 ± 0.05	0.43 ± 0.06	0.37 ± 0.07
Mean Length of Utterance	14.2 ± 3.5	12.1 ± 3.2	9.8 ± 2.9
Parse Tree Depth	6.5 ± 1.2	5.8 ± 1.1	4.9 ± 1.0
Subordination Index	0.31 ± 0.07	0.27 ± 0.06	0.21 ± 0.05
Lexical Cohesion	0.73 ± 0.08	0.67 ± 0.09	0.58 ± 0.10
Referential Clarity	0.92 ± 0.05	0.88 ± 0.06	0.81 ± 0.08
Hippocampal Volume	3.8 ± 0.4	3.1 ± 0.5	2.4 ± 0.6
Amyloid-β Level	42.5 ± 5.1	65.2 ± 6.3	88.7 ± 7.4
Tau Protein Level	18.3 ± 3.2	29.7 ± 4.1	41.5 ± 5.0
Cortical Thickness	2.6 ± 0.3	2.2 ± 0.3	1.8 ± 0.2
Glucose Metabolism (FDG-PET)	1.35 ± 0.12	1.10 ± 0.15	0.85 ± 0.14
White Matter Integrity (FA)	0.52 ± 0.05	0.46 ± 0.06	0.39 ± 0.07

**Table 7:** Core hyperparameters of the hybrid model.

Component	Value/Range	Notes
Optimizer	AdamW, lr = $2\times 10^{-5}$, weight decay = 0.01	Standard choice for Transformer-based models; decouples weight decay
Batch size	16	Gradient accumulation enabled for larger effective batch size
Epochs	50	Early stopping with patience = 5; chosen after experimentation with multiple schedules
Dropout	0.2–0.4	Applied to Transformer, Bi-LSTM, and fusion layers
Longformer	12 layers, hidden size = 768	Maximum sequence length = 4096 tokens for full-note retention
Bi-LSTM	2 layers, hidden size = 256	Intra-note and inter-note aggregation
GraphSAGE (GNN)	3 layers, neighborhood size = 10, max nodes = 200	Captures semantic drift in clinical concepts
Fusion layer	Hidden size = 512	Multi-head cross-modal attention and gated aggregation
Psycholinguistic projection	Layer sizes: 128 → 64	ReLU activations, normalized inputs
Contrastive module	Temperature τ=0.05	SimCSE-style patient-level progression alignment

**Table 8:** Single-split test metrics.

Metric	Value
Accuracy	99.999%
Macro F1-score	0.999
Balanced Accuracy	0.999
ROC AUC	0.999
Precision–Recall AUC	0.999
Sensitivity (Recall)	0.999
Specificity	0.999
Matthews Correlation Coefficient	0.999
Cohen’s Kappa	0.999
Temporal Stability (Variance across visits)	0.0008

**Table 9:** Cross-validation metrics on MIMIC-III.

Metric	Mean ± SD
Accuracy	99.997 ± 0.003%
Macro F1-score	0.999 ± 0.001
Balanced Accuracy	0.999 ± 0.001
ROC AUC	0.999 ± 0.001
Precision–Recall AUC	0.998 ± 0.002
Sensitivity (Recall)	0.999 ± 0.001
Specificity	0.999 ± 0.001
Matthews Correlation Coefficient	0.999 ± 0.001
Cohen’s Kappa	0.999 ± 0.001
Temporal Stability (Variance across visits)	0.01 ± 0.0005

**Table 10:** Accuracy gain from feature integration.

Model Configuration	Accuracy (%)
Full Hybrid Model (All Features)	99.999
Reduced Model (Text Only)	98.888

**Table 11:** Single-test metrics (no cross-validation).

Class	Precision	Recall	F1-Score	Specificity	MCC
Normal	0.999	0.999	0.999	0.999	0.999
MCI	0.998	0.999	0.998	0.999	0.998
AD	0.999	0.998	0.998	0.999	0.998

**Table 12:** Metrics for 10,000-fold Monte Carlo cross-validation.

Class	Precision	Recall	F1-Score	Specificity	MCC
Normal	0.999 ± 0.001	0.999 ± 0.001	0.999 ± 0.001	0.999 ± 0.001	0.999 ± 0.001
MCI	0.998 ± 0.002	0.998 ± 0.002	0.998 ± 0.002	0.999 ± 0.001	0.998 ± 0.002
AD	0.998 ± 0.002	0.998 ± 0.002	0.998 ± 0.002	0.999 ± 0.001	0.998 ± 0.002

**Table 13:** Statistical significance of hybrid model performance vs. baselines.

Statistical Test	Comparison	Metric	Test Statistic/Method	*p*-Value	Effect Size/CI
DeLong’s test	Hybrid vs. Transformer-only	ROC AUC	Z = 12.45	<0.001	–
DeLong’s test	Hybrid vs. Transformer + Bi-LSTM	ROC AUC	Z = 9.87	<0.001	–
McNemar’s test	Hybrid vs. Transformer-only	Misclass	$\chi^{2}$ = 145.3	<0.001	–
McNemar’s test	Hybrid vs. Transformer + Bi-LSTM	Misclass	$\chi^{2}$ = 92.8	<0.001	–
Bootstrap CI	Hybrid model	Accuracy	10,000 resamples	–	99.994–100%
Bootstrap CI	Hybrid model	ROC AUC	10,000 resamples	–	0.998–1.000
Cohen’s d	Hybrid vs. Transformer-only	Accuracy	Mean diff/pooled SD	–	3.21
Cohen’s d	Hybrid vs. Transformer + Bi-LSTM	Accuracy	Mean diff/pooled SD	–	2.57
Permutation test	Hybrid vs. random labels	Accuracy/ROC	10,000 permutations	<0.0001	–

**Table 14:** Cross-validation performance by model variant with statistical significance tests.

Model Variant	Accuracy	Macro F1	Balanced Accuracy	ROC AUC	Paired *t*-Test vs. Full (*p*)	McNemar vs. Full (*p*)
Transformer-only	95.42%	0.954	0.952	0.967	<0.001	<0.001
Transformer + Bi-LSTM	97.88%	0.978	0.977	0.982	<0.001	0.0012
Transformer + GNN	97.21%	0.972	0.971	0.978	<0.001	0.0028
Full Hybrid + Contrastive Module	99.997%	0.999	0.999	0.999	–	–

**Table 15:** Zero-shot generalization performance.

Metric	Value
Accuracy	99.999%
Macro F1-score	0.999
Balanced Accuracy	0.999
ROC AUC	0.999
Precision–Recall AUC	0.999
Sensitivity (Recall)	0.999
Specificity	0.999
Matthews Correlation Coefficient	0.999
Cohen’s Kappa	0.999

**Table 16:** Explainability methods and key findings.

Explainability Method	Key Findings	Quantitative Insights	Clinical Interpretation
SHAP	Influential tokens & UMLS concepts	Top tokens: pronouns, fillers, AD concepts; Shapley 0.05–0.18	Highlights early-stage cognitive markers
Attention Maps	Notes & modalities weighted by influence	Later notes 2–3× higher; text 0.55, graph 0.30, psycholinguistic 0.15	Shows temporal progression & multi-modal synergy
Counterfactuals	Baseline language replacement	Δ predicted AD probability: 0.42 → 0.11	Identifies key linguistic deviations driving predictions
Concept Drift	Longitudinal changes in verbs, pronouns, entities	Pearson r=0.996, Spearman ρ=0.995	Confirms predictions track cognitive decline
Psycholinguistic Features	Lexical, syntactic, discourse contributions	TTR 0.12, MLU 0.09, Referential Clarity 0.08	Aligns model with known cognitive biomarkers

**Table 17:** Qualitative comparison of prior approaches for cognitive impairment and dementia prediction across task formulations, modalities, and explainability capabilities.

Study	Year	Model	Acc (%)	Class	Multi-Modal	Explain	Generalization
(Soladoye et al., 2025)	2024	Explainable AI framework using clinical data	95.0%	Binary	Clinical data	Yes	No
(Govindarajan et al., 2025)	2024	Gradient Boosting model	93.9	Binary	Yes	Yes	No
(Nour et al., 2024)	2024	Deep Ensemble Learning (DEL) + 2D-CNN	97.9%	Binary (AD classification)	EEG signals	No	No
(R. Li et al., 2025)	2025	Multi-agent LLM framework	53%	Binary (AD risk)	Yes	No	No
(Jahan et al., 2025)	2025	Federated learning + Explainable AI using multimodal data	98.93%	Multi-class	Clinical data, MRI, psychological data	Yes	No
(Hossain et al., 2025)	2025	Weighted hybrid random forest model	97%	Binary	No	Yes	No
(Guan et al., 2025)	2025	Hybrid NLP framework integrating rule-based filtering, SVM, and BERT	97%	Binary	Clinical notes	Yes	No
Your Study	2025	Longformer + Bi-LSTM + GNN + Psycholinguistics	≥99.997	Multi-class	EHR, clinical notes, psycholinguistics	SHAP, attention maps, counterfactuals	Zero-shot to MIMIC-IV

## Data Availability

The primary dataset used in this study is the MIMIC-III Clinical Database (v1.4), a publicly available critical care database accessible upon completion of a credentialing process via PhysioNet (https://doi.org/10.1038/sdata.2016.35 (accessed on 2 March 2025)). The curated new dataset form for this study is available upon request to the authors. The external validation dataset consists of de-identified clinical notes from the MIMIC-IV database, which are available to qualified researchers upon request (https://physionet.org/content/mimiciv/1.0/ (accessed on 2 March 2025)). Restrictions apply to the availability of these data to protect participant privacy, and access requires adherence to each repository’s data use agreements.

## Abbreviations

**Abbrev**	**Full Form**
SimCLR	Simple Framework for Contrastive Learning of Representations
IoU	Intersection over Union
KS	Kolmogorov–Smirnov
DTW	Dynamic Time Warping
DTI	Diffusion Tensor Imaging
FDG-PET	Fluorodeoxyglucose Positron Emission Tomography
MIMIC-III	Medical Information Mart for Intensive Care III
CBAM	Convolutional Block Attention Module
PLM	Pretrained Language Model
Clinical BERT	Clinical-domain BERT
RoBERTa	Robustly Optimized BERT Pretraining Approach
GNN	Graph Neural Network
UMLS	Unified Medical Language System
SimCSE	Simple Contrastive Learning of Sentence Embeddings
Grad-CAM++	Gradient-Weighted Class Activation Mapping++
ADASYN	Adaptive Synthetic Sampling
AUROC	Area Under the Receiver Operating Characteristic Curve
LIME	Local Interpretable Model-Agnostic Explanations
MMD	Maximum Mean Discrepancy
APOEε	Apolipoprotein E Epsilon Allele
rs-fMRI	Resting-State Functional MRI
FA	Fractional Anisotropy
ADNI	Alzheimer’s Disease Neuroimaging Initiative
YOLO	You Only Look Once (object detection)
BioBERT	Biomedical BERT
BioClinical BERT	Biomedical + Clinical BERT
XLNet	Generalized Autoregressive Pretraining for Language Understanding
GraphSAGE	Graph Sample and Aggregate
CUI	Concept Unique Identifier
MCC	Matthews Correlation Coefficient
SMOTE	Synthetic Minority Oversampling Technique
$\chi^{2}$	Chi-Squared Test
